# Splinted and Nonsplinted Crowns with Different Implant Lengths in the Posterior Maxilla by Three-Dimensional Finite Element Analysis

**DOI:** 10.1155/2018/3163096

**Published:** 2018-09-03

**Authors:** Cleidiel Aparecido Araujo Lemos, Fellippo Ramos Verri, Joel Ferreira Santiago Junior, Victor Eduardo de Souza Batista, Daniel Takanori Kemmoku, Pedro Yoshito Noritomi, Eduardo Piza Pellizzer

**Affiliations:** ^1^Department of Dental Materials and Prosthodontics, Araçatuba School of Dentistry, State University (UNESP), Araçatuba, SP, Brazil; ^2^Department of Health Science, Sacred Hearth University–USC, Bauru, SP, Brazil; ^3^Department of Prosthodontics, University of Western São Paulo–UNOESTE, Presidente Prudente, SP, Brazil; ^4^Information Tecnology Center Renato Archer, Campinas, SP, Brazil

## Abstract

The aim of this study was to evaluate stress distribution in the implants/components and bone tissue for splinted and nonsplinted prostheses with different lengths of implants using three-dimensional finite element analysis. Six models from the posterior maxillary area were used in simulations. Each model simulated three Morse taper implants of 4.0 mm diameter with different lengths, which supported metal-ceramic crowns. An axial load of 400 N and an oblique load of 200 N were used as loading conditions. Splinted prostheses exhibited better stress distribution for the implants/components, whereas nonsplinted prostheses exhibited higher stress in the first molar under axial/oblique loading. Implant length did not influence stress distribution in the implants/components. In cortical bone tissue, splinted prostheses decreased the tensile stress in the first molar, whereas nonsplinted prostheses were subjected to higher tensile stress in the first molar; implant length had no influence on stress distribution. Within the limitations of this study, we conclude that splinted prostheses contributed to better stress distribution in the implant/abutment and cortical bone tissue; however, the reduction in the implant length did not influence the stress distribution.

## 1. Introduction

Dental implants are considered to be a predictable treatment option for rehabilitation of edentulous patients and have demonstrated high success rates, reestablishing masticatory function and aesthetics [1]. Maintenance of bone tissue around the implant, however, is still considered to be a significant challenge, primarily for implants placed in atrophic maxillary posterior regions [2].

Generally, the available bone tissue present in this area is insufficient for the placement of longer implants [3]. In these cases, supplementary surgical procedures, such as maxillary sinus augmentation, have been suggested for placement of dental implants [4]. However, this surgery is associated with a higher risk for surgical complications, morbidity, and a higher cost of treatment [5]. Therefore, short implants are considered to be a simple and effective rehabilitation alternative in cases of limited bone quantity in the maxillary posterior region [5].

Some studies have reported that short implants exhibit unfavorable biomechanical behavior compared with standard implants [6], leading to a lower survival rate for short implants [3]. However, some authors have suggested the splinting of short implants with longer implants to reduce biomechanical risks [7]. This may contribute to increasing the survival rate of short implants when placed in the maxillary posterior region [8]. However, no consensus has been reached because some studies have reported that splinted prostheses do not influence the stress distribution when compared with nonsplinted prostheses [9, 10].

Accordingly, the present study aimed at evaluating the stress distribution on implant/abutment and bone tissue in terms of splinted crowns with different lengths of Morse taper implants in fixed implant-supported prostheses. The null hypotheses were as follows: splinted and nonsplinted crowns have similar stress distribution on implant/abutments and bone tissue, and there are no differences in the stress distribution in terms of different lengths of Morse taper implants.

## 2. Materials and Methods

This research was developed considering three factors: crown design (nonsplinted or splinted crowns), implant length (11.5; 10; 8.5, and 7 mm), and loading (axial and oblique). Six models were used in simulations (Table 1). Each model simulated a bone block of the maxillary posterior region (right first premolar to right first molar), with trabecular bone surrounded by a 1 mm cortical bone layer obtained using InVesalius software (CTI Renato Archer, Campinas, SP, Brazil) and surface simplification with Rhinoceros 3D 4.0 software (NURBS Modeling for Windows, Seattle, WA, USA).

**Figure 1:**
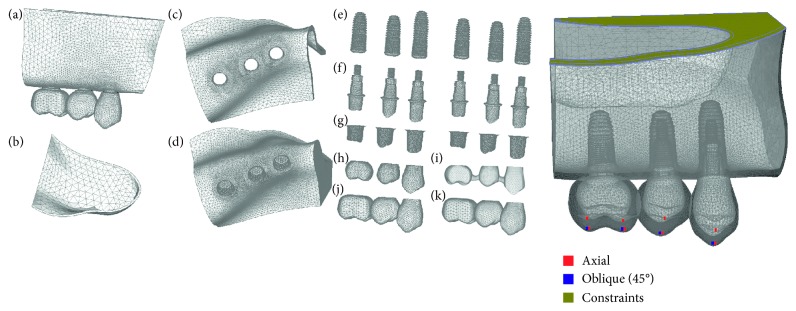
Schematic view of meshes of structures, load conditions, and restrictions. (A) 3D model; (B) cortical bone of maxillary sinus; (C) cortical bone; (D) trabecular bone; (E) Morse taper implants; (F) abutment with screw fixation; (G) cement; (H) framework of nonsplinted prosthesis; (I) framework of splinted prosthesis; (J) crown of nonsplinted prosthesis; (K) crown of splinted prosthesis.

The implant design was obtained by simplification of the original design of the Morse taper implant (Torq, Conexão Sistemas de Prótese Ltda, Aruja, SP, Brazil), measuring 4 mm in diameter and implant lengths of 11.5, 10, 8.5, and 7 mm. All Morse taper implants were simulated with 1 mm subcrestal placement. The crown designs were obtained from an artificial tooth (Odontofix Indústria e Comércio de Material Odontológico Ltda., Ribeirão Preto, SP, Brazil) using a 3D scanner (MDX-20; Roland DG, São Paulo SP, Brazil). The designs were exported to Rhinoceros 4.0 CAD software for simplification and modeling. The implant-supported crowns were simulated using a cement connection, with a cement layer thickness of 50 *µ*m and variation of splinted and nonsplinted crowns. The indexed abutment used was the universal long-cast abutment (UCLA), which was simplified using Rhinoceros 4.0 software while maintaining similarity to the original abutment (Figure 1).

After modeling, all geometries were exported to discretization in the finite element software FEMAP 11.2 (Siemens PLM Software Inc., Santa Ana, CA, USA) for preprocessing to obtain meshes of tetrahedral parabolic solid elements for all structures. The mechanical properties of each simulated material were attributed to the meshes using values established in previous studies (Table 2) [11–14]. All materials were considered isotropic, homogeneous, and linearly elastic.

For this study, symmetrical welds were considered for all contacts, except for the abutment/implant contact and interproximal crowns of nonsplinted models, for which symmetric contact was simulated. Boundary conditions were fixed in the *x*, *y*, and *z* axes, simulating fixation of the maxilla (cortical and trabecular), whereas all other model surfaces were unrestricted. The nonlinear applied force was 400 N axially, with 50 N for each internal slope of the cusps, and 200 N obliquely, with 50 N at each lingual internal slope of the cusps (Figure 1).

All models were exported to the NeiNastran 11.0 software (Noran Engineering Inc., Westminster, CA, USA). The processing analysis was performed using a workstation (Hewlett-Packard Development Co) with the following characteristics: Intel Xeon Processor X3470, 16 GB RAM, and 2 TB of storage. Results were exported to FEA software (FEMAP v11.1.2; Siemens PLM Software Inc) to create maps of stress on implant/abutment/crown and bone tissue. Von Mises analysis was used to assess the stress distribution in implant/abutment/crown, whereas the cortical bone tissue was evaluated using maximum principal stress and are distinguished between tensile stresses (positive values) and compressive stresses (negative values). The unit of measurement for both analyses in the present study was megapascals (MPa) [15, 16].

## 3. Results

### 3.1. Von Mises Stress (Implants/Abutment/Crown)

**Figure 2:**
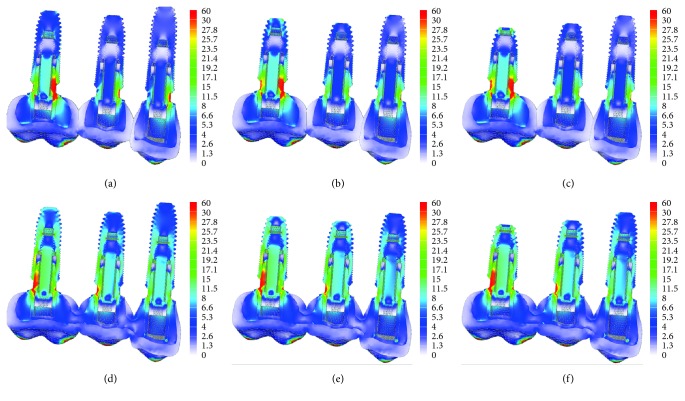
The von Mises stress distribution on implant/abutment in the axial loading. (A) Nonsplinted with 11.5 mm, 10 mm, and 10 mm; (B) nonsplinted with 10 mm, 8.5 mm, and 8.5 mm; (C) nonsplinted with 10 mm, 8.5 mm, and 7 mm; (D) splinted with 11.5 mm, 10 mm, and 10 mm; (E) splinted with 10 mm, 8.5 mm, and 8.5 mm; (F) splinted with 10 mm, 8.5 mm, and 7 mm).

Under axial loading, nonsplinted crowns exhibited higher stress concentration on mesial of the first molar abutment, whereas splinted crowns contributed to share the stress between the implants, decreasing the stress in the mesial but increasing the stress in the distal abutment of the first molar. Furthermore, the stress distribution in splinted crowns was concentrated throughout the implant, whereas in the nonsplinted crowns, the stress was concentrated in the cervical area. No differences between implant lengths were observed, regardless of crown design (Figure 2).

**Figure 3:**
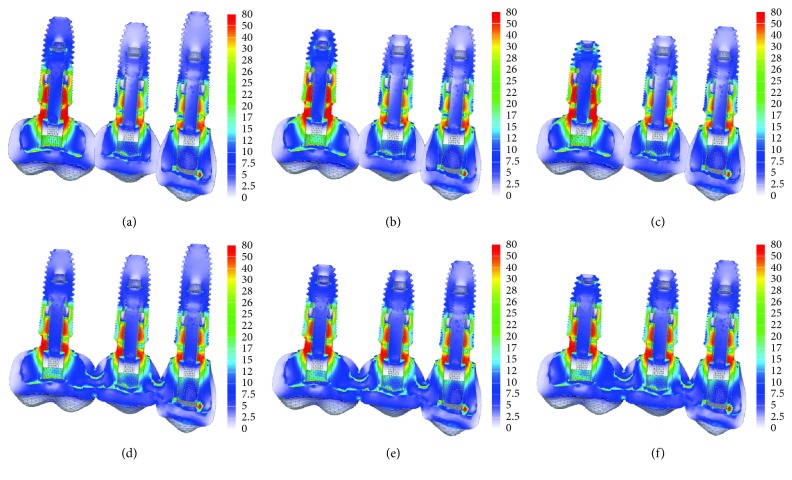
The von Mises stress distribution on implant/abutment in the oblique loading. (A) Nonsplinted with 11.5 mm, 10 mm, and 10 mm; (B) nonsplinted with 10 mm, 8.5 mm, and 8.5 mm; (C) nonsplinted with 10 mm, 8.5 mm, and 7 mm; (D) splinted with 11.5 mm, 10 mm, and 10 mm; (E) splinted with 10 mm, 8.5 mm, and 8.5 mm; (F) splinted with 10 mm, 8.5 mm, and 7 mm).

Under oblique loading, the splinted crowns contributed to a decrease in stress in the abutment and cervical/middle region of the implant in the first molar region when compared with nonsplinted crowns; however, the length of the implants did not influence the stress distribution (Figure 3).

### 3.2. Maximum Principal Stress (Cortical Bone Tissue)

**Figure 4:**
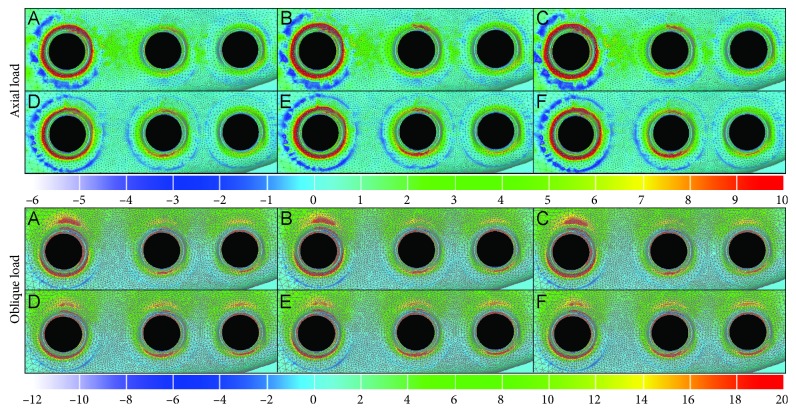
Superior view of maximum principal stress in the cortical bone tissue under axial and oblique loading. (A) Nonsplinted with 11.5 mm, 10 mm, and 10 mm; (B) nonsplinted with 10 mm, 8.5 mm, and 8.5 mm; (C) nonsplinted with 10 mm, 8.5 mm, and 7 mm; (D) splinted with 11.5 mm, 10 mm, and 10 mm; (E) splinted with 10 mm, 8.5 mm, and 8.5 mm; (F) splinted with 10 mm, 8.5 mm, and 7 mm).

The axial loading showed less tensile stress on cortical bone tissue, mainly in the first molar region when compared with oblique loading. The splinted crowns contributed to stress distribution, decreasing tensile stress in the first molar region; however, a slight increase in tensile stress on the second premolar region was observed when compared with nonsplinted crowns. In the oblique loading, the splinted crowns reduced tensile stress in the first molar region when compared with nonsplinted crowns. Regarding the length of implants, no difference in the stress distribution on cortical bone tissue was noticed, independent of loading conditions (Figure 4).

## 4. Discussion

This study assessed the effect of splinted crowns and implant length of Morse taper because this connection type exhibits better biomechanical behavior than other connections [17, 18], contributing to bone preservation [19, 20] and lower complications rates [21]. Furthermore, cemented crowns were simulated in this study because they exhibit better biomechanical behavior with Morse taper implants [16] and contribute to greater preservation of the bone tissue, compared with the screwed crown [22].

The first null hypothesis in this study was rejected because a reduction in the stress distribution on implant/abutments and bone tissue for splinted crowns was observed. These results are consistent with that of previous studies, which also reported that splinted crowns improve the sharing of stress with adjacent implants in other implants connections [7, 23]. The advantage of splinted crowns in sharing stress with other implants may be explained by the rigid union of components, thus enabling the stress distribution between implants [7]. This may contribute to decreasing the stress on implants that are subject to high masticatory forces, such as those placed in the molar region. The higher stress in the molar may be attributed to increases in the occlusal table with four cusps [16]. In this context, the splinted crowns of implants with a greater occlusal table is recommended because it may promote better stress distribution, thus decreasing the complication rates such as loosening/fracture screw fixation and abutment [7], and the risk for resorption of cortical bone tissue [8], in the maxillary posterior region, which exhibit higher risk of implant failure [24].

Although a difference in stress distribution was observed for splinted crowns, this difference was subtler for bone tissue, and this may have been influenced by an internal connection system that provides better dissipation of tensions due to the high connection stability [18, 25], independent of the crown design. Clelland et al. [26] reported that splinted crowns did not exhibit significant differences when compared with the nonsplinted implants; however, splinted crowns contributed to more uniform distribution of stresses. Thus, indications for nonsplinted cemented crowns under Morse taper implants are also feasible due to easy access in the interproximal area, enabling better hygiene [27] and patient adaption, which in turn positively contribute to improve the quality of life [28].

Regarding the length of the implants, no significant difference was observed in stress distribution, especially in the first molar region with the greater variation in length (10, 8.5, and 7 mm). Thus, the second null hypothesis was accepted. Some factors may contribute to similarity in stress in short implants compared with standard implants, such as the implant connection used [17, 18], cemented crowns [22, 29], and subcrestal implants [30] which may contribute to the reduction of stress on the structures. Furthermore, the differences in the implants length were small (11.5, 10, 8.5, and 7 mm). Thus, further studies investigating extra-short implants (< 7 mm) is recommended because lower survival rates for extra-short implants, compared with longer-length implants, have been reported [3].

Although there is a difference in the stress distribution of splinted crowns when compared with that for nonsplinted crowns in implant/abutments and bone tissue, this difference does not exceed the theoretical limits of stresses for dental implants and bone tissue established in the literature [18, 31]. Thus, both crown designs may be recommended for clinicians.

Finite element analysis has been considered in biomechanical studies to verify variables not yet consolidated by clinical studies; however, it has limitations because it is a computational analysis [18]. This type of analysis favors the biomechanical understanding of structures in an individualized manner, which may be used to investigate important structures for the longevity of treatment in dental implant/abutments and bone tissue. The results of this analysis may be cautiously extrapolated to the clinic [32] and subsequently used to complement future randomized trials.

## 5. Conclusion

Within the limitations of this study, it may be concluded that splinted crowns favor the stress distribution by reducing the stress in the implant/abutment and cortical bone tissue. However, the reductions in the implant length did not influence the stress distribution.

## Figures and Tables

**Table 1:** Specifications of the models.

Models	Implant	Crown design	Length of implants	Nodes/elements
Model A	Morse taper (*Ø* 4.0 mm)	Nonsplinted	11.5 mm (1° PM)	1970105/1049429
			10 mm (2° PM)	
			10 mm (1° M)	
Model B			10 mm (1° PM)	1909453/999937
			8.5 mm (2° PM)	
			8.5 mm (1° M)	
Model C			10 mm (1° PM)	1886171/983572
			8.5 mm (2° PM)	
			7 mm (1° M)	
Model D		Splinted	11.5 mm (1° PM)	1988751/1062241
			10 mm (2° PM)	
			10 mm (1° M)	
Model E			10 mm (1° PM)	1928099/1012749
			8.5 mm (2° PM)	
			8.5 mm (1° M)	
Model F			10 mm (1° PM)	1904817/996376
			8.5 mm (2° PM)	
			7 mm (1° M)	

**Table 2:** Mechanical properties of the simulated materials.

Structures	Modulus of elasticity (GPa)	Poisson ratio (*ν*)	Reference
Trabecular bone	1.37	0.30	[11]
Cortical bone	13.7	0.30	[12]
Titanium (implant and abutment)	110.0	0.35	[12]
Metal alloy	206.0	0.33	[13]
Feldspathic porcelain	82.8	0.35	[14]
Zinc phosphate cement	22.4	0.35	[13]

## Data Availability

The data used to support the findings of this study are included within the article.
